# Elastomer-Based Visuotactile Sensor for Normality of Robotic Manufacturing Systems

**DOI:** 10.3390/polym14235097

**Published:** 2022-11-24

**Authors:** Islam Mohamed Zaid, Mohamad Halwani, Abdulla Ayyad, Adil Imam, Fahad Almaskari, Hany Hassanin, Yahya Zweiri

**Affiliations:** 1 Advanced Research and Innovation Center, Khalifa University, Abu Dhabi 127788, United Arab Emirates; 2 Department of Aerospace Engineering, Khalifa University of Science and Technology, Abu Dhabi 127788, United Arab Emirates; 3 School of Engineering, Technology, and Design, Canterbury Christ Church University, Canterbury CT1 1QU, UK

**Keywords:** elastomer, tactile sensor, robotic, manufacturing, hole quality, drilling, deburring

## Abstract

Modern aircrafts require the assembly of thousands of components with high accuracy and reliability. The normality of drilled holes is a critical geometrical tolerance that is required to be achieved in order to realize an efficient assembly process. Failure to achieve the required tolerance leads to structures prone to fatigue problems and assembly errors. Elastomer-based tactile sensors have been used to support robots in acquiring useful physical interaction information with the environments. However, current tactile sensors have not yet been developed to support robotic machining in achieving the tight tolerances of aerospace structures. In this paper, a novel elastomer-based tactile sensor was developed for cobot machining. Three commercial silicon-based elastomer materials were characterised using mechanical testing in order to select a material with the best deformability. A Finite element model was developed to simulate the deformation of the tactile sensor upon interacting with surfaces with different normalities. Additive manufacturing was employed to fabricate the tactile sensor mould, which was chemically etched to improve the surface quality. The tactile sensor was obtained by directly casting and curing the optimum elastomer material onto the additively manufactured mould. A machine learning approach was used to train the simulated and experimental data obtained from the sensor. The capability of the developed vision tactile sensor was evaluated using real-world experiments with various inclination angles, and achieved a mean perpendicularity tolerance of 0.34°. The developed sensor opens a new perspective on low-cost precision cobot machining.

## 1. Introduction

Robotics, one of the key pillars of industry 4.0, has been increasingly used in aerospace manufacturing for grasping, painting, and precision machining operations such as drilling, deburring, fastening, and inspection [1]. Compared to computer numerical control (CNC) machining, robotics can provide better accuracy, consistency, cost-effectiveness, and flexibility with their ability to carry out various tasks. In addition, the robotics’ degree of freedom motion allows them to access difficult-to-reach areas and parts [1,2,3]. Aeroplane structures are typically made from titanium and aluminium alloys. Machining of these materials can generate geometrical stress concentration spots affecting their properties, especially the fatigue life of the structure [4]. Drilling is an important machining process of aircraft manufacturing, as high-quality holes are required to produce bolted and riveted joints needed during aircraft assembly. More than 40 million holes per year are drilled in an aircraft production line to assemble aircraft wings [5].

Drilling high-strength aluminium and titanium alloys typically results in trapped heat in holes that are difficult to dissipate due to the generated friction and chip formation between the drill tool and the aerospace structure. In addition, most drilled holes form burrs on both sides of the structure, which promote cracks’ formation and propagation and reduce fatigue life [5,6,7,8,9]. To remove holes’ burrs, a deburring post-processing operation is required for the hole edge finishing. Perpendicularity (normality) is an important geometrical tolerance of drilled holes. Perpendicularity error of drilled holes must be less than 2° to ensure that the mechanical connections through rivets or bolts are resistant to cyclic loads [10,11,12]. These strict requirements make it hard to utilise cobots in drilling and deburring processes compared to conventional or robotic drilling operations. Additive manufacturing technologies (AM), also known as three-dimensional printing, play notable roles in various research and industrial applications. This technology is capable of manufacturing parts with complex geometries in a short time compared to conventional techniques [13,14]. The design and material freedom of AM have enabled the technology to become rapidly popular over the past few years. This allows the development of several AM technologies, not only to fabricate models and functional parts, but also in the development of sensors and actuators [15]. The sensors, actuators, and fixtures developed through AM play a significant role in enhancing the precision and repeatability of a manufacturing process in the Aerospace industry. One such sensing technology that directly benefits from the AM is vision-based tactile sensing, which directly addresses the perpendicularity challenges in robot-based automated manufacturing.

Vision-based tactile sensing (VBTS) is an advanced perception technology that uses vision systems to monitor the deformation of an elastomer upon contact with different surfaces, which allows it to sense the topographic information of these surfaces. VBTS offers multiple advantages over conventional tactile sensors, including high spatial resolution, design simplicity, and low instrumentation requirements [16]. These advantages have motivated the research community to investigate the application of VBTS in a plethora of functions, such as object manipulation [17,18,19], slip detection [20,21], contact force measurement [22,23,24], pose estimation [25] and texture recognition [26]. Fang et al. [27] established a dual modal fingertip elastomer tactile sensors for grasping and recognizing objects. The sensor was employed with a reflective object with markers that can measure the object texture and the force distribution. A similar study introduced by Hu et al. [28] developed an improved elastomer sensor for grasping objects using a sputtered aluminium, which acts as markers with a mask of flexible printed circuit board. The camera and the developed algorithm were able to recognise the markers and hence the deformed elastomer with real time speed. Kamiyama et al. [29] developed a 40 mm thick elastomer sensor to measure the contact forces. This enabled a wider range of the measurement of tactile sensors. However, it was difficult to implement it to the current robotic systems due to its bulky design. Therefore, thinner thickness elastomer geometry were developed in several studies. Yang et al. [30] and Sui et al. [31] introduced thinner thickness tactile sensors for surface sensing and slip detection. Due to their widespread application, several studies in the literature have focused on the simulation of VBTS sensors to support design optimisation and Sim2Real learning. Gomes et al. [32] introduced a synthetic VBTS system to support a Sim2Real learning process, which employed filters to construct the height map with different intensities. Sferrazzai et al. [33] developed a simulation of VBTS that generates synthetic images of spherical particles in the sensor’s elastomer to reconstruct the 3D distribution of the contact forces. However, the sensor requires a large set of data to accurately sense the surface. Wang et al. [34] developed an RGB simulator for a VBTS system that had the ability not only to sense surfaces but also to grasp objects. However, the sensor was only tested in simulation, and no hardware validation or testing was presented in that study.

To date, no study in the literature has considered the design nor simulation of VBTS 94 systems to address the perpendicularity (normality) challenges of robotics in the aerospace manufacturing industry. This paper demonstrates in detail the step-by-step process to develop a novel vision-based tactile sensor that provides the required perpendicularity measurements for drilling, deburring, and machining processes. Additionally, this paper outlines the Finite Element Analysis (FEA) and simulation of this novel sensor that can be used for design optimisation and Sim2Real learning. Finally, this paper evaluates the capabilities of the presented FEA simulations to accurately depict the sensor’s response in experimental settings, and introduces a Sim2Real deep learning model that leverages data from simulations to estimate perpendicularity within the specified tolerances of 2°. The remainder of this paper is organised as follows: Section 2 details the fabrication, design, material testing and Finite Element Simulation of our novel vision-based tactile sensor for perpendicularity measurements. Section 3: The evaluation and validation process we conduct is twofold. First, we compare and quantify the deformation patterns between simulation and experiments across different contact conditions. Second, we evaluate the Sim2Real capabilities of our FEA simulations by developing and training a deep learning model purely on simulated data, and assessing the model’s performance experimentally. Finally, Section 4 summarises the findings and results of this paper.

## 2. Methodology

**Figure 1:**
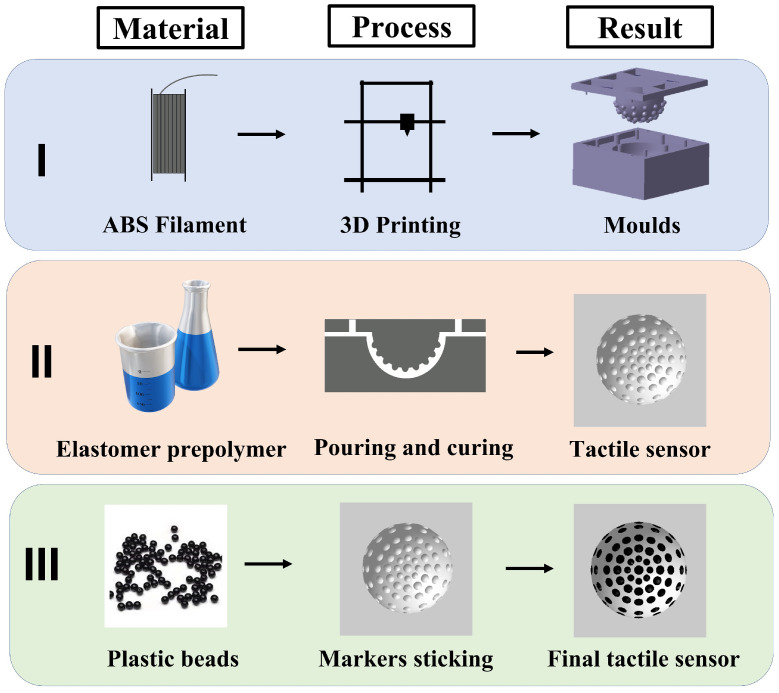
The fabrication process of the tactile sensor consists of three main stages: (**I**) ABS material is used in an FDM 3D printer to fabricate the upper and lower moulds. (**II**) Liquid silicon material colored by silicon pigments is poured and cured in the 3D-printed mould. (**III**) Internal markers consisting of plastic beads are precisely sticked in the required location.

The development of the vision-based tactile sensor started with a characterization process of three different elastomers’ materials aiming to select the most flexible one to achieve a high-sensitivity material to the applied deformation. After choosing the material, a CAD mould was designed with a cavity representing the geometry of the tactile sensor. The CAD model was 3D printed and used to prepare the elastomer tactile sensor by casting and curing the elastomer prepolymer. To create the markers, spherical plastic beads were used and placed on cavities created on the inner surface of the sensor. A schematic diagram of the process is shown in Figure 1.

### 2.1. Sensor Materials and Fabrication

Silicone-based elastomers are semi-inorganic polymers with a silicon-oxygen chain and two hydrocarbon groups. Polydimethylsiloxane (PDMS) is a popular silicon-based elastomer that is not only used for micro/nanofabrication [35,36,37] but also used by several researchers in developing tactile sensors [38,39,40]. Other silicon-based elastomers have been recently investigated for the development of tactile sensors. Three different elastomers; namely Dragon Skin™30, Ecoflex™00-30, and Ecoflex™00-50; have been considered in this study. Those elastomers are platinum-catalysed silicones with favourable properties for tactile sensors, such as soft, firm, stretchy, and simple to prepare.

**Figure 2:**
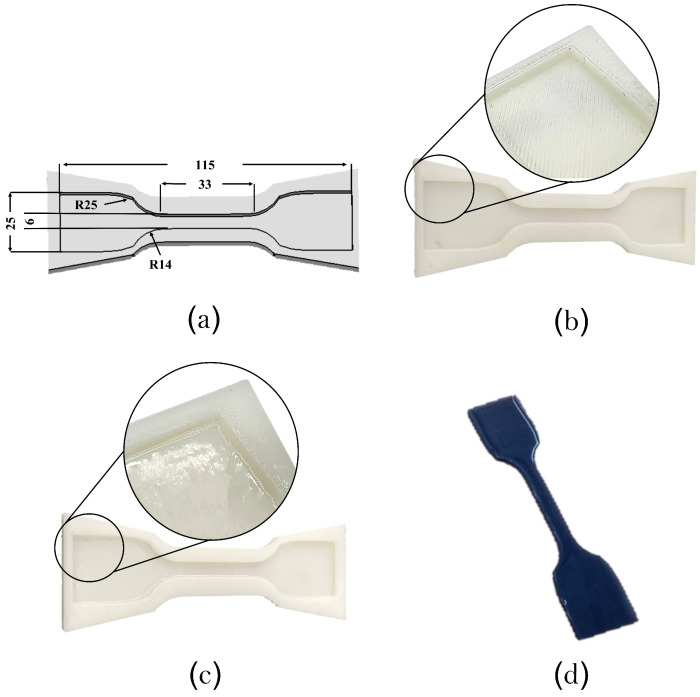
The fabrication process of the elastomer testing specimen. (**a**) CAD model of the specimen mould showing the specimen dimensions in mm. (**b**) 3D-printed mould showing the poor surface finish. (**c**) The specimen mould after acetone curing improves the surface quality. (**d**) Elastomer sample after curing and readying for tensile testing.

The characterisation of the elastomers was carried out using tensile testing experiments according to ASTM D-412C standard using a Birmingham universal mechanical testing machine (Instron 6800 series, Khalifa University). The 3D-printed mould representing the geometry of the tensile specimens was carried out using Zortax M200 3D printer, as in Figure 2a,b. Acrylonitrile butadiene styrene (ABS) filament was used to print the samples with a layer thickness of 0.29 and infill density of 100%. Several factors can influence the tensile testing of the samples, in particular, the surface roughness of the gauge length. Poor surface quality act as a stress riser, which affects the mechanical properties of the samples. A surface finish post-processing was carried out using an acetone bath to improve the surface quality of the mould, as shown in Figure 2c. The three elastomer slurries were prepared using Dragon Skin™30, Ecoflex™00-30, and Ecoflex™00-50 prepolymers (Smooth-On, Inc., Macungie, PA, USA). All materials were supplied as two parts (1A1:1B1), mixed using a mechanical stirrer for 2 min, and left under a vacuum for 5 min to remove air bubbles. Next, the slurry was cast onto the tensile sample moulds and left to cure for 4 and 16 h for ™and Dragon Skin™elastomers, respectively. Elastomer samples were peeled off and used for tensile testing, Figure 2d.

**Figure 3:**
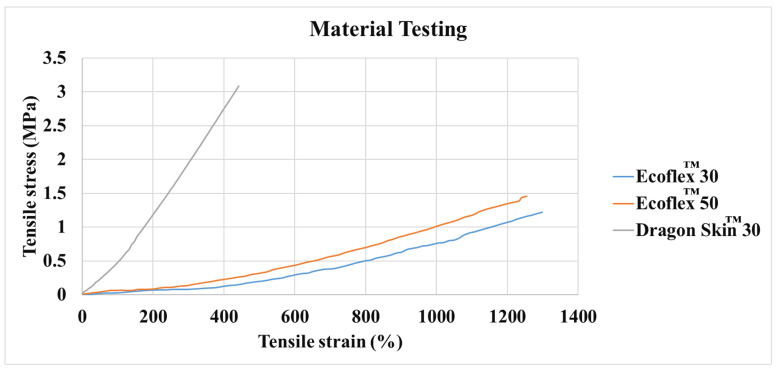
Stress–strain curve of the three materials.

Table 1 lists the mechanical properties of the tested samples, whereas Figure 3 shows the stress–strain of Dragon Skin™30, Ecoflex™00-30, and Ecoflex™00-50 samples. Dragon Skin™30 samples have higher mechanical strength and Young’s modulus compared to Ecoflex™00-30, and Ecoflex™00-50. On the other hand, Ecoflex™00-30 was found to have the highest ductility and flexibility. High flexibility means the sensor will be more deformable, hence, improving the measurement accuracy. Therefore, Ecoflex™00-30 elastomer was selected for the development of the sensor.

**Figure 4:**
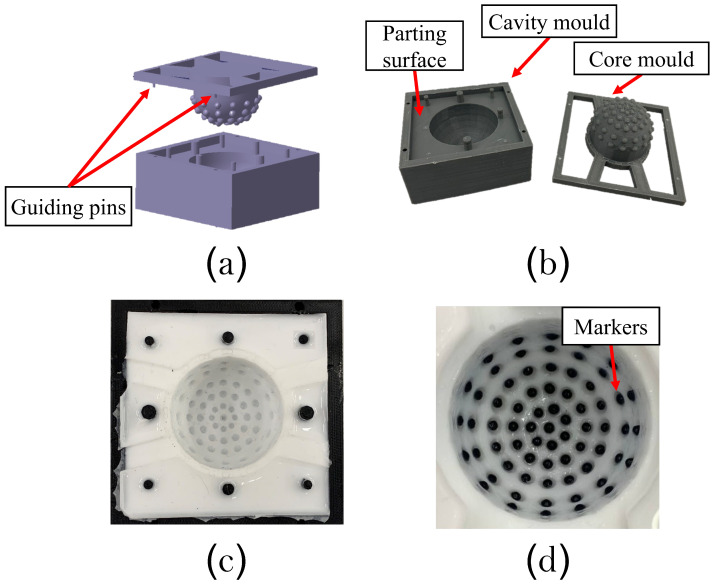
The fabrication process of the elastic tactile surface. (**a**) CAD model of the tactile core mould. (**b**) The 3D printed core mould. (**c**) The core mould with the cured material. (**d**) The final sensor after sticking the markers to the internal pockets.

The fabrication of the vision-based tactile sensor starts with creating a CAD model, which represents the sensor’s hemispherical geometry. A mould design representing the cavity and core of the sensor model was also designed with a parting plan at the planar surface of the hemisphere. In addition, guidance pins were included in the core part to ensure easy assembly, Figure 4a shows the cavity and core design. Next, the mould was 3D printed with the same parameters as the tensile test samples, see Figure 4b. Ecoflex™00-30 parts A and B were mixed using a mechanical stirrer for two minutes, and left under vacuum to remove air bubbles. The mix was then coloured with silicon pigments and poured inside the cavity mould after degassing the material in a vacuum chamber. This process ensures a smoother surface with no trapped air bubbles. The core mould is then assembled and left to cure, as shown in Figure 4c. For better deformation detection and visibility, different coloured plastic beads are then embedded on the internal surface of the tactile body. The markers are precisely stuck to the pockets on the internal sensor surface by a thin transparent layer of silicon material, as shown in Figure 4d. The design features of the tactile sensor such as the materials for the mould and the senor, the size of the sensors, the markers size, and the number of the markers are summarised in Table 2.

### 2.2. Experimental Setup

**Figure 5:**
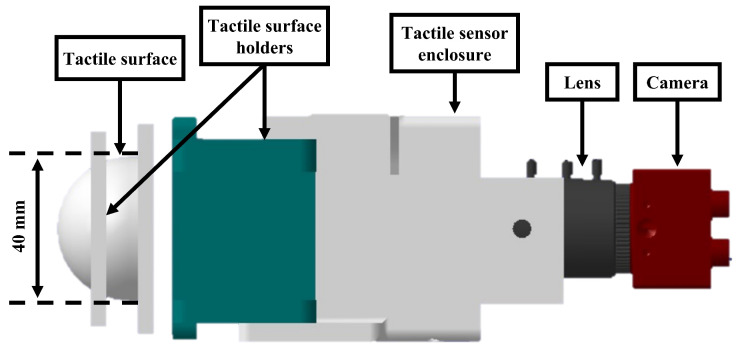
The sensor compact design consists of a 3D-printed enclosure that holds a camera facing the backside of the tactile surface to capture the markers’ arrangement.

The proposed vision-based tactile sensor consists of a housing structure made by 3D printing, the camera and the tactile surface, as illustrated in Figure 5. Markers are arranged on the inside surface of the tactile surface. The internal markers are black plastic beads with colors that contrast with the tactile surface for better visibility. The markers are distributed radially over the hemisphere at distinct angles. A camera is positioned towards the tactile surface’s backside side in order to observe the markers. The hemispherical surface of the sensor’s elastic surface deforms significantly when force is applied, altering the magnitude and direction of the markers’ movement.

**Figure 6:**
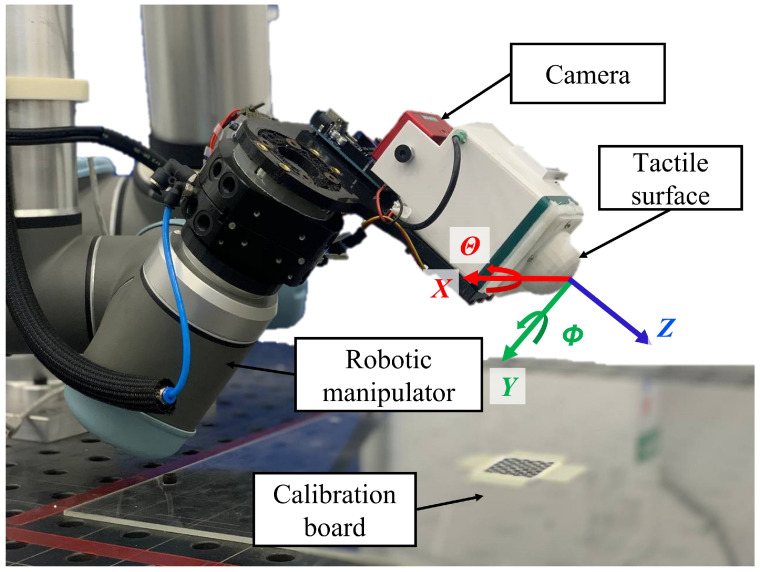
The experimental setup with the tactile sensor mounted on UR10 robotic manipulator showing the tactile surface frame that is precisely calibrated with the calibration board.

The sensor was mounted on a UR10 robot manipulator that was programmed to press the surface up against the calibrated flat surface in different relative poses. For each pose, an image is sampled along with the pose measurements after achieving contact with the surface. Figure 6 shows the data experimental setup together with the sensor frame. Using a DAVIS346 camera with a 346 × 280 resolution, the dataset was recorded by adjusting the roll θ angle, where the robotic manipulator moves to the contact surface for each angle and pushes the tactile surface on the flat contact surface along its normal vector *Z*.

### 2.3. Finite Element Modelling

**Figure 7:**
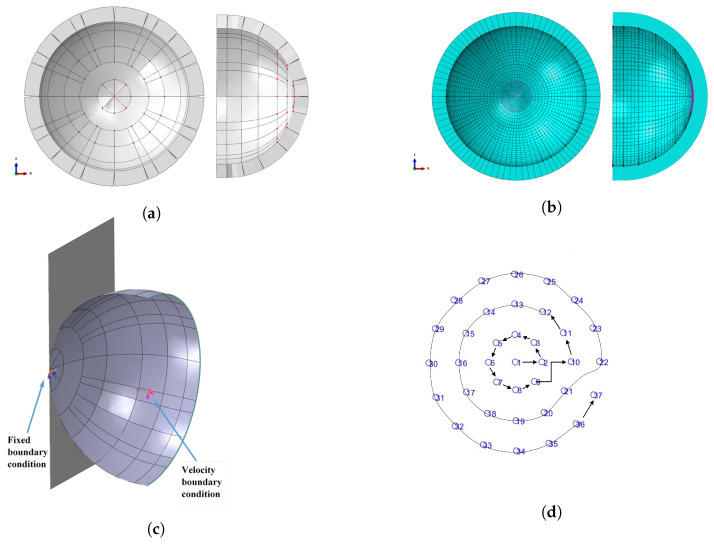
(**a**) Model markers; (**b**) model meshing; (**c**) model boundary conditions; (**d**) markers’ sorting.

A Finite element model was created using ABAQUS CAE (Dassault Systèmes, Vélizy-Villacoublay, France) to characterise the sensor deformation when in contact with a wall at various inclination angles, as shown in Figure 7. Firstly, the CAD geometries of both the hemispherical tactical sensor and the wall were imported to ABAQUS analysis system to develop the FE simulation. The sensor model was assumed to move towards the rigid wall with a constant speed of 1 cm/s. A general contact relation was employed to specify the interfacial contact between the sensor and the wall. The wall was modelled as an analytical rigid body and was meshed as a single node across the geometry of the plane, whereas the elastomer sensor was modelled using 3D deformable shell elements with a thickness of 4 mm.

The sensor model was assumed to have a uniform thickness, neglecting the variation due to markers’ fabrication and the plastic beads. The shell surface that carries the markers was assigned to the inner surface, considering the shell element thickness starts from the shell surface outward. The shell has been partitioned into a number of cells that make a group of nodes at the corners (intersections). The majority of the cells correspond to sensor markers, where the movement of the nodes represents the movement of the centre of the markers. Figure 7c shows the sensor cells with the location of the markers. The markers are hemispherical in shape, and stacked and stuck to the inner surface of the hemispherical sensor. This configuration affects the outer circular patterns by the side projection, where most of the markers are not perfectly projected to the camera plane, which shifts between the markers’ centres in the simulation and the experiment. Figure 7b illustrates the way of sorting the markers and the nodes to be easily compared. The central marker is considered the first point, followed by the marker on the positive direction of horizontal axes in the next circular pattern, then moving counterclockwise.

Figure 7c shows the boundary conditions of the model. The first boundary condition is located at the central point of the rigid wall, which is constrained to the rigid wall. The second boundary condition is applied to represent the relative movement of the sensor, and a fixed velocity is specified to the positive direction of Z assigned to central reference point. This point is constrained to the circle at the base of the sensor. Mesh refinements were applied to the shell structure using a global hexahedral S4R (4-node doubly curved 218 shell, reduced integration, hourglass control, and finite membrane strains) mesh of general sizing 0.001 mm, resulting in 3189 nodes and 3144 elements in the sensor geometry with a 220 maximum deviation factor of 0.1. Figure 7b shows the meshed sensor geometry.

™00-30 stress–strain data were employed to determine constants, see Figure 3, and obtain the material model used in the FEA. An isotropic linear elastic material model was used to model the material, where Young’s Modulus (E=0.039 MPa), Density (ρ=1050 kgm^−3^) and a Poisson’s ratio of 0.42. The relationship between Young’s modulus, shear modulus and Poisson’s ratio is shown in Equation (1).

(1) E=2G(1+v)

where *E* is Young’s modulus, *G* is the shear modulus and *v* is Poisson’s ratio.

**Figure 8:**
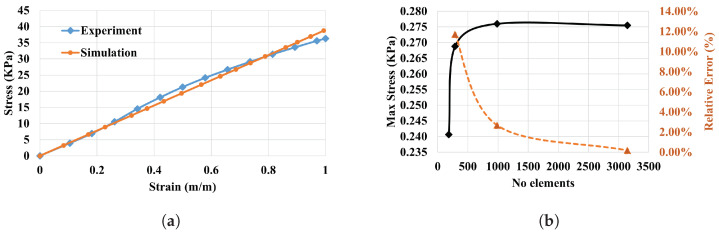
(**a**) Stress–strain diagram of Ecoflex™00-30; (**b**) mesh sensitivity analysis.

Figure 8a shows a comparison between the simulated material model and the obtained experimental data. The figure indicates a good agreement between the two models.

Mesh sensitivity analysis was carried out to optimise the element size at which a stable and accurate solution can be realised at a reasonable computational time. The results of the mesh sensitivity have been presented in Figure 8b. As shown, the meshing error is stabilised at about 3144 elements.

The markers are hemispherical in shape, where they are stacked and stuck to the inner surface of the hemispherical sensor. This configuration makes the outer circular patterns affected by the side projection, where most of the markers are not perfectly projected to the camera plane, which makes a shift between the centers of the markers in the simulation and the experiment. Figure 7d illustrates the way of sorting the markers and the nodes to be easily compared to each other. The central marker is considered as the first point followed by the marker in the positive direction of horizontal axes in the next circular pattern, and then moving counterclockwise.

## 3. Results and Discussion

**Figure 9:**
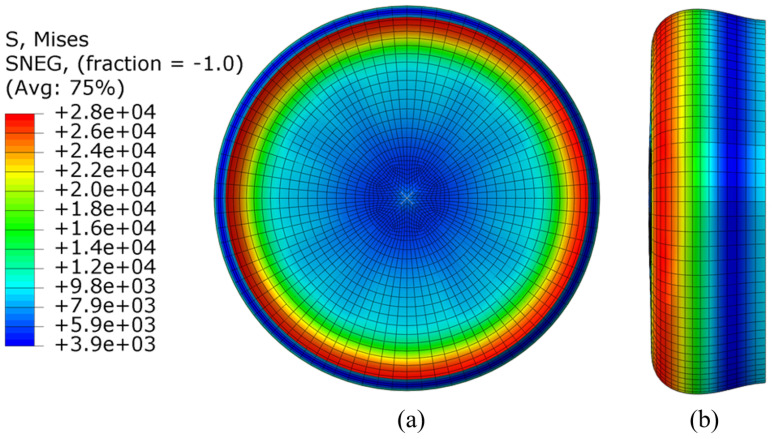
Von-mises stress analysis of the elastomer sensor under 1 cm pressing stroke of 1 cm: (**a**) Top view; (**b**) side view.

The finite element model was first checked against the material properties to ensure that the developed stresses and strain due to the sensor deformation is within the implemented material model. Figure 9 shows the developed Von mises stress of the model from the side and top view. As shown, maximum stresses were found as a ring-shaped area on the outer circumference of the elastomer sensor. This is the area that is in contact with the vertical plan at the end of the 1 cm stroke. The maximum stresses were located in the outer layers of the sensor due to the high bending stresses of elastomer deformation. A maximum stress of 28 KPa was obtained over the aforementioned pressing stroke, which is less than the range of the material model, as shown in Figure 8a.

**Figure 10:**
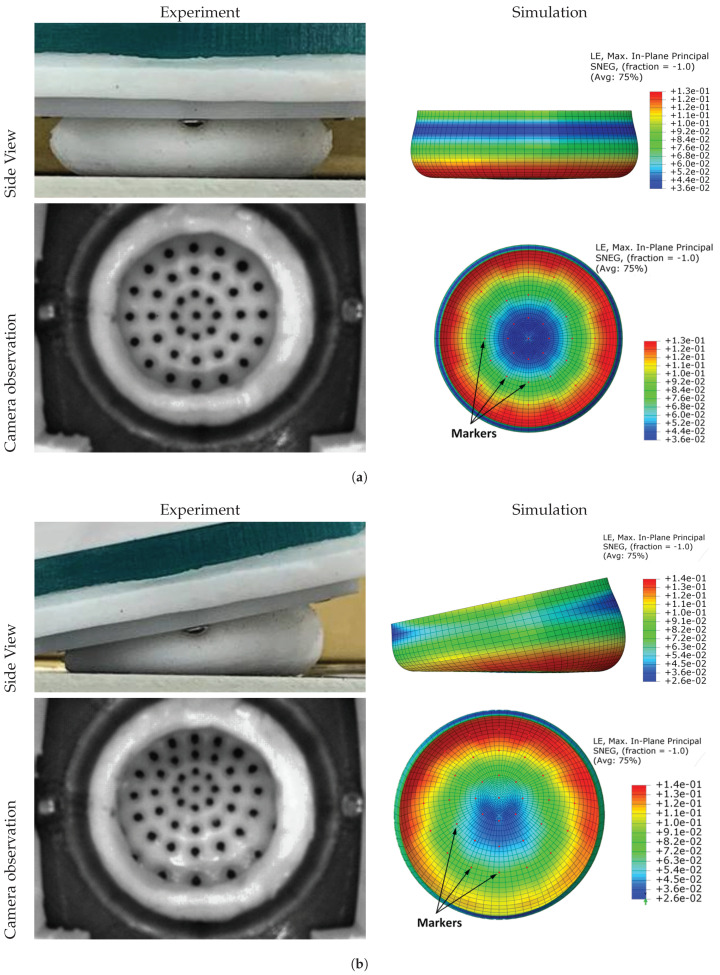
Actual and simulated results of the sensor deformation at different angles. (**a**) at angle 0°. (**b**) At angle 12°.

The finite element model was validated by comparing the simulated and experimental results of the tactile sensor markers, which were captured using the camera fitted to the cobot manipulator. Figure 10 compares the actual and simulated deformation of the tactile sensors at 0° and 12° inclination angles. Overall, the figure shows a good agreement between the experimental and the simulation mode. The elastomer sensor was deformed vertically at 0° inclination angle, as shown in Figure 10a. The simulation results of the side and the top views show symmetry in both the deformation and the locations of markers. Figure 10b shows the elastomer deformation at an inclination angle of 12°. The highest deformation was found at the opposite side of the inclination direction. Furthermore, the wall compressed the elastomer at the direction of the inclination angle, which resulted in denser markers, whereas looser markers were found in the opposite direction of the wall inclination angle. The markers’ movements can be observed as a two-dimensional motion in both *X* and *Y* directions, which can be captured and processed by the camera images and a deep-learning model.

The average error between the experimental and the simulation data was calculated as the differences between the centre points of the associated markers divided by the number of points in the *X* and *Y* directions. Over time, the error was calculated as the square difference between their locations, as shown in Equations (2) and (3). This concept had been used by other researchers. In one of the studies, the markers were grouped and the average of the translation and rotation movements was calculated in [41]. Li et al. have proposed a continuous marker pattern (CMD), where they applied a fitting function to make the continuous representation of the deformed surface [42].

(2) $$E_{U,n}\left(S\right)=\frac{\int_{s=0}^{S}{\left(U_{sim,n}\left(s\right)-U_{exp,n}\left(s\right)\right)}^{2}ds}{S}$$

(3) $$E_{V,n}\left(S\right)=\frac{\int_{s=0}^{S}{\left(V_{sim,n}\left(s\right)-V_{exp,n}\left(s\right)\right)}^{2}ds}{S}$$

(4) $$\mathrm{Total Accumulative Error}=\Sigma_{n=1}^{N}\sqrt{\frac{E_{U,n}+E_{V,n}}{N}}$$

where: $E_{U,n},E_{V,n}$ are the error in *U* and *V* directions respectively, and $U_{sim,n}$, $U_{exp,n}$, $V_{sim,n}$, $V_{exp,n}$ are the fitted functions of the simulation and experiment in *U* and *V* directions. *S* is the travelled depth of 10 mm. *N* is the total number of 37 nodes.

**Figure A1:**
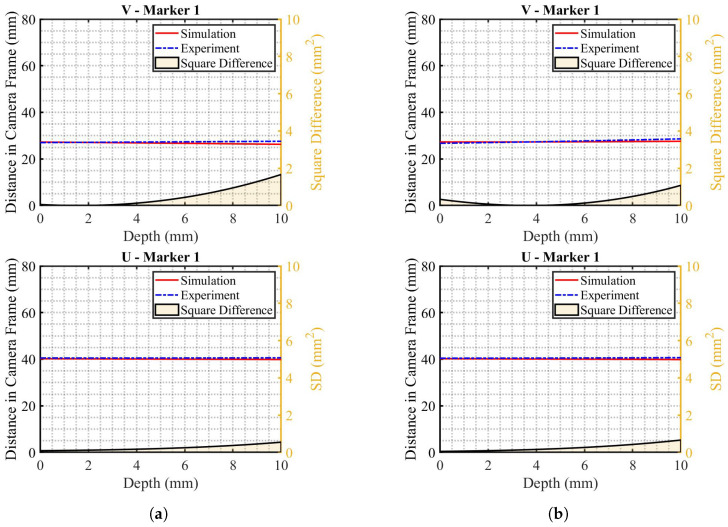

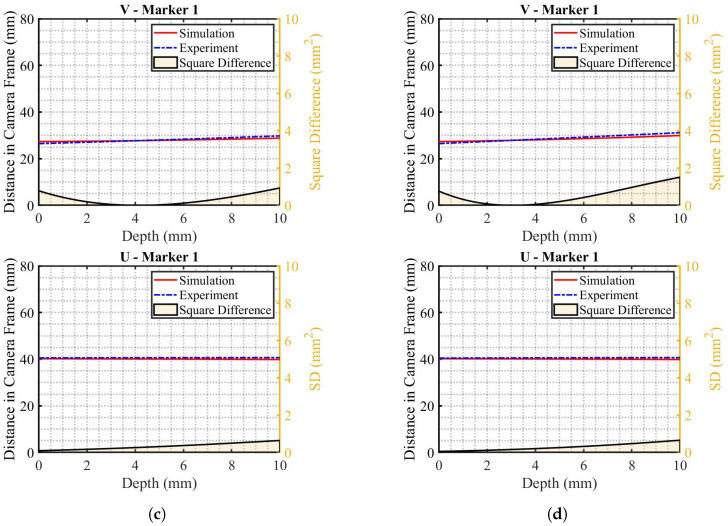
The deformation in U and V directions and the error between simulation and experiment over a pressing stroke of 10mm for different angles. (**a**) Normal configuration of 0°. (**b**) Inclined configuration of 4°. (**c**) Inclined configuration of 8°. (**d**) inclined configuration of 12°.

Table 3 lists the error in the markers’ locations at wall inclinations of 0°, 4°, 8°, and 12°. As shown in the table, there is a good agreement between the simulated and experimental data, resulting from the low errors across all of the elastomer markers. The average error at a wall angle of 0° and 4° was found to be lower than both wall angles of 8° and 12°. On the other hand, the highest error was obtained at marker 27 (the outer circle) at an inclination angle of 0°. This is because some markers at the outer circles may be difficult to be captured accurately by the camera. The deformation and error values between the simulation and experimental data of all markers in the *U* and *V* directions when the elastomer sensor is deformed by 10 mm stroke are shown in Table A1 and Figure A1.

### Deep Learning for Sim2Real

The last criteria by which we assess the FEA simulations is in terms of sim2real transferability to train deep learning (DL) models for the prediction of contact angle. The application of deep learning using neural networks has been established in many applications including recognizing complex patterns and images [43]. To that end, we develop a fully connected neural network and train it on the simulation data described in Section 2.3. We then test the network’s accuracy and precision on the experimental data described in Section 2.2. This section details the network architecture, hyperparameter tuning, and the testing results of our Sim2Real deep learning model.

**Figure 11:**
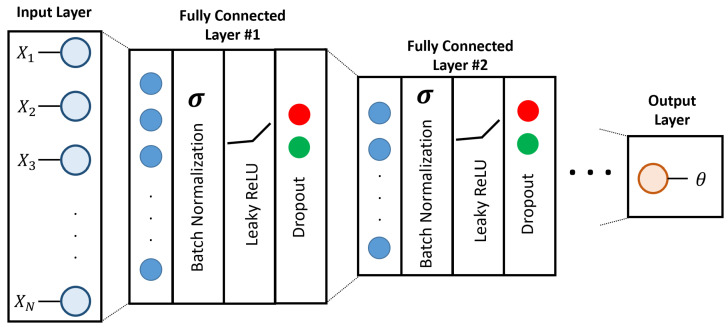
The architecture of the contact angle estimation DL model. The input is a vector of size 74 that corresponds to the pixel coordinates of the markers on the elastomer material. The model consists of $N_{dense}$ fully connected hidden layers, each followed by batch normalization, dropout and a leakyReLU activation function. The output layer represents the estimated contact angle.

The objective of our DL model is to find the mapping Γ:X→θ, where the input *X* is the pixel coordinates of the markers on the tactile sensor’s elastomer, and the output θ is the contact angle. We employ and train fully-connected networks with the architecture shown in Figure 11 to solve this mapping. Fully-connected deep learning networks are powerful tools for pattern recognition, where high-level features are learned from lower-level ones to serve a classification or regression function [43]. Our network consists of $N_{dense}$ fully connected layers each having a width of $N_{width}^{j}$, where $j\in [1,N_{dense}]$. Batch normalization is applied to the output of each layer to accelerate training and to add a slight regularization effect [44]. Each fully connected layer is followed by a leaky ReLU activation function for faster convergence [45]. The dropout is then applied to prevent overfitting and prompt a noise rejection behavior [46]. The final layer contains a single unit corresponding to the predicted contact angle θ.

The DL model is trained purely on simulation data. Training data includes the contact angles in the range [0°,12°] at varied contact depths from 0 to 10 mm. Each data sample consists of the pixel coordinates in *U* and *V* for each of the 37 markers, resulting in an input vector of size 74. The initial simulation dataset consists of 143 different samples. To enhance the generalization and Sim2Real performance of the DL model, we augment the initial dataset to generate additional data. We perform augmentation by randomly perturbing the pixel coordinates of the elastomer’s markers. From the analysis in Section 3, we note that the maximum mismatch in pixel coordinates between experimental data and FEA simulations was two pixels. As such, in our augmentation process, we perturb the marker’s location of simulation data in both *U* and *V* directions by *N* pixels, where *N* is a uniformly sampled number in the range [−2,2]. It must be noted that the perturbation of each marker is performed independently from other markers within the same data sample. Upon augmentation, the overall number of data samples becomes 1430. We divide this dataset into training, validation, and testing subsets on a 60%-20%-20% basis.

The DL model was trained using the ADAM optimization algorithm [47], with the Mean-Squared-Error being selected as the loss function. We performed a hyperparameter optimization process to select the best learning hyperparameters. The hyperparameters included in this optimization process are summarized in Table 4, along with their considered ranges and optimal values. The optimal hyperparameter values were selected based on their performance on the held-out validation set.

The performance of the developed DL model for estimating the contact angle was evaluated on an unseen subset of the simulation data, in addition to unseen experimental data. Table 5 summarizes the obtained results in terms of Mean Absolute Error (MAE), standard deviation and maximum errors. Results demonstrate an exceptional Sim2Real performance of the developed DL model, with the MAE being 0.34 on the experimental data, even though the model has never been exposed to experimental data during the training process. These results conform with the perpendicularity requirements of 2° in the aerospace manufacturing industry. Most importantly, these results verify the accuracy of our FEA simulations in capturing the physical deformation characteristics of elastomers in VBTS; as the DL model trained on simulated deformations does not exhibit any significant loss in performance when evaluated on experimental data. These promising results open a new paradigm for developing and testing VBTS algorithms purely on FEA simulations, without the need for expensive and time consuming experiments. This can be extended to functions beyond just contact angle measurements, such as force distribution estimation, where obtaining ground truth force distributions on experimental data demands an expensive and complicated sensing system. Furthermore, the proper match between simulation and experimentation enables the design optimization of VBTS systems using simulation data only, where such simulations can be used to find the optimal geometry and material of the elastomers in these novel sensors. Although Ecoflex™00-30 showed a good performance as a visuotactile sensor due to its low elastic modulus, other materials such as hydrogels are receiving significant attention and demand in the field of flexible devices [48] which makes them worth exploring in future work.

## 4. Conclusions

In this paper, we presented a detailed procedure to design, simulate, validate, and fabricate a low-cost elastomer for a vision-based tactile sensor to support cobots for the machining of aerospace structures. The tactile sensor can measure the normality information of surfaces using a sensitive elastomer object, with internal markers relying on the deformation of the markers captured using a camera and processed using a deep learning approach. First, the mechanical characteristics of Dragon Skin, Ecoflex™00-50, and Ecoflex™00-30 elastomer materials were evaluated. Ecoflex™00-30 elastomer showed a lower elastic modulus and higher ductility, which made it a suitable elastomer for the proposed tactile sensor. A hemispherical finite element model of the tactile sensor with internal markers was developed using shell elements in contact with a rigid wall with different normalities. The tactile sensor was fabricated by the direct casting of the elastomer polymer onto an additively manufactured mould. The fabricated sensor demonstrated a good agreement with the simulation model. In addition, the deep learning model was trained using the simulation and experimental results of the sensor deformation. The deep learning model prediction of the contact angle shows exceptional sim2real transferability. The performance of the sensor shows a mean normality tolerance of 0.13°, which satisfies the requirements of aircraft manufacturing.

## Figures and Tables

**Table 1:** The mechanical properties of the measured elastomer materials.

Elastomer	Ultimate Strength (MPa)	Young’s Modulus (MPa)	Ductility (Strain %)
Dragon Skin™30	3.118	0.907	490
Ecoflex™00-30	1.115	0.039	1290
Ecoflex™00-50	1.480	0.099	1250

**Table 2:** Design features of the tactile sensor elastic material and internal markers.

Parameter	Value
Tactile surface material	Ecoflex™00-30 (E = 0.039 MPa) (rubber (Hardness: Shore 00-30))
Tactile surface size	40 mm diameter
Markers’ material	plastic
No. of markers	37
The diameter of markers	2.5 mm
Size of the sensor enclosure (L × H × W)	20 cm × 12 cm ×10 cm
Sensors’ 3D-printed enclosure material	ABS

**Table 3:** Error between simulation and experiment for all the markers.

Node Number	Error (mm)				Node Number	Error (mm)			
	0°	4°	8°	12°		0°	4°	8°	12°
1	0.297	0.279	0.297	0.311	21	0.326	0.349	0.368	0.369
2	0.338	0.339	0.35	0.348	22	0.346	0.372	0.398	0.382
3	0.326	0.318	0.334	0.326	23	0.35	0.354	0.381	0.361
4	0.363	0.325	0.324	0.323	24	0.329	0.327	0.355	0.326
5	0.326	0.31	0.319	0.317	25	0.371	0.348	0.363	0.345
6	0.308	0.29	0.306	0.322	26	0.397	0.36	0.366	0.355
7	0.248	0.258	0.276	0.284	27	0.425	0.373	0.361	0.358
8	0.275	0.284	0.3	0.324	28	0.387	0.34	0.331	0.336
9	0.326	0.33	0.35	0.355	29	0.358	0.321	0.318	0.34
10	0.338	0.348	0.366	0.359	30	0.308	0.28	0.285	0.335
11	0.323	0.323	0.342	0.329	31	0.257	0.277	0.297	0.346
12	0.352	0.343	0.362	0.344	32	0.223	0.257	0.275	0.321
13	0.367	0.332	0.338	0.33	33	0.277	0.292	0.288	0.306
14	0.394	0.354	0.358	0.359	34	0.269	0.304	0.315	0.315
15	0.349	0.321	0.329	0.339	35	0.325	0.355	0.368	0.369
16	0.277	0.269	0.28	0.309	36	0.34	0.375	0.394	0.393
17	0.263	0.266	0.283	0.315	37	0.338	0.373	0.401	0.393
18	0.224	0.25	0.263	0.285	**min**	**0.223**	**0.25**	**0.263**	**0.284**
19	0.239	0.271	0.292	0.307	**max**	**0.425**	**0.375**	**0.401**	**0.393**
20	0.29	0.316	0.333	0.337	**avg**	**0.32**	**0.318**	**0.331**	**0.337**

**Table 4:** The hyperparameters range of the DL model, and the corresponding optimal values.

Parameter	Parameter Range	Optimal Value
Number of hidden layers $N_{dense}$	[1,2,3,4,5]	5
Width of hidden layers $N_{width}$	[16,32,64,128,256,512,1024]	$N_{width}^{1-5}=\left[1024,512,128,64,32\right]$
Dropout coefficient	[0.0,0.5]	0.5
Batch size	[64,128,256,512]	256
Number of epochs	[64,128,256,512,1024]	128
Base learning rate	[0.001,0.01,0.1]	0.01

**Table 5:** Experimental and simulation performance evaluation of the contact angle results using the developed DL model. The model was trained purely on simulation data, but shows exceptional sim2real transferability.

Dataset	MAE	Standard Deviation	Max Error
Simulation	0.41°	0.37°	1.65°
Experimental	0.34°	0.31°	1.33°

## Appendix A. Tables and Figures

**Table A1:** Error between simulation and experiment for all the markers.

Node Number	Error (mm)							
	0°		4°		8°		12°	
	U	V	U	V	U	V	U	V
1	1.509	1.749	1.509	1.361	1.767	1.507	1.629	1.945
2	2.939	1.294	2.971	1.271	3.1	1.43	2.876	1.618
3	2.61	1.311	2.538	1.209	2.714	1.422	2.501	1.439
4	2.718	2.146	2.561	1.34	2.493	1.393	2.314	1.556
5	2.092	1.84	2.154	1.395	2.296	1.47	2.12	1.587
6	1.578	1.933	1.502	1.602	1.711	1.758	1.67	2.168
7	0.957	1.326	1.078	1.376	1.26	1.562	1.014	1.97
8	1.634	1.161	1.742	1.25	1.866	1.456	2.034	1.848
9	2.754	1.169	2.815	1.224	3.061	1.462	2.953	1.718
10	3.148	1.085	3.176	1.314	3.367	1.594	3.197	1.567
11	2.769	1.087	2.682	1.186	2.885	1.435	2.689	1.309
12	3.43	1.146	3.309	1.032	3.487	1.35	3.229	1.151
13	3.037	1.939	2.925	1.153	3.059	1.167	2.819	1.2
14	3.124	2.614	3.062	1.579	3.247	1.493	2.972	1.784
15	1.842	2.653	1.952	1.85	2.125	1.873	1.965	2.293
16	0.426	2.42	0.718	1.965	0.862	2.043	0.748	2.795
17	0.739	1.82	0.781	1.83	0.852	2.104	0.831	2.836
18	0.521	1.338	0.652	1.656	0.703	1.855	0.692	2.305
19	0.849	1.26	1.157	1.555	1.389	1.769	1.503	1.989
20	1.724	1.387	2.008	1.679	2.358	1.735	2.394	1.796
21	2.811	1.123	3.019	1.482	3.316	1.686	3.326	1.704
22	3.524	0.905	3.564	1.559	3.919	1.951	3.909	1.49
23	3.763	0.77	3.607	1.023	3.858	1.51	3.71	1.124
24	3.087	0.925	2.851	1.104	2.996	1.673	2.825	1.103
25	3.749	1.334	3.566	0.914	3.777	1.093	3.465	0.93
26	3.95	1.869	3.807	0.998	3.932	1.016	3.667	0.993
27	3.498	3.185	3.452	1.684	3.635	1.175	3.32	1.424
28	2.142	3.392	2.141	2.126	2.394	1.671	2.168	2.022
29	0.567	4.167	0.644	3.169	1.012	2.729	0.892	3.376
30	0.957	2.541	0.887	2.024	0.79	2.216	0.989	3.162
31	1.315	1.138	1.279	1.556	1.183	2.074	1.328	3.111
32	0.831	1.006	0.844	1.593	0.682	2.112	0.783	3.024
33	1.26	1.574	1.137	2.009	0.906	2.157	0.924	2.548
34	0.709	1.965	0.995	2.426	1.448	2.227	1.521	2.15
35	1.756	2.141	2.057	2.609	2.577	2.44	2.864	2.185
36	2.502	1.784	2.836	2.377	3.371	2.386	3.677	2.024
37	3.108	1.117	3.345	1.816	3.824	2.127	3.96	1.742
sum	79.931	63.615	81.322	59.295	88.222	64.119	85.475	70.987
min	0.426	0.77	0.644	0.914	0.682	1.016	0.692	0.93
max	3.95	4.167	3.807	3.169	3.932	2.729	3.96	3.376
avg	2.16	1.719	2.198	1.603	2.384	1.733	2.31	1.919
